# Medicinal Plants Used to Treat Human and Livestock Ailments in Basona Werana District, North Shewa Zone, Amhara Region, Ethiopia

**DOI:** 10.1155/2022/5242033

**Published:** 2022-04-14

**Authors:** Moa Megersa, Nigussie Tamrat

**Affiliations:** Department of Biology, School of Natural and Computational Sciences, Madda Walabu University, P.O. Box 247, Robe, Ethiopia

## Abstract

This study was conducted on medicinal plants used for the treatment of human and livestock ailments in Basona Werana District, North Shewa Zone, Amhara Region. Data were collected through semi-structured interviews, field walk observation, preference, and direct matrix ranking with randomly and purposefully selected informants. A total of 80 respondents (46 men and 14 women) and 20 (16 men and 4 women) traditional healers participated in this study. A total of 76 plant species distributed in 75 genera and 45 families were collected and identified. Of the 76 medicinal plants collected from the study area, 85.5% were used to treat human ailments. The Lamiaceae came out as a leading family with 8 (10.5%) species followed by Asteraceae and 7 (9%) medicinal plant species each, while Solanaceae followed with 6 (7.8%) species. The majority of medicinal plants were collected from wild habitat and accounted for 56 plant species (73.6%). The result of growth form analysis showed that herbs constituted the highest proportion of medicinal plants represented by 33 species (43.4%), followed by shrubs with 30 species (39.4%) and trees with 10 species (13.1%). The medicinal plants were administered through oral, which accounts for 54 species (48.1%), followed by dermal with 38 species (33.9%) and nasal with 9 species (8%), respectively. Leaves were the most frequently used plant parts for the preparation of traditional herbal medicines in the study area. Crushing was the widely used preparation method (33.9%) followed by pounding (16%). *Cucumis ficifolius* A. Rich. was the most preferred plant used to treat stomachache. Phytochemical and pharmacological studies of this type of plant are recommended to get the most out of the plant.

## 1. Introduction

Medicinal plants are playing a vital role in the treatment of human and livestock ailments. It is estimated that up to 80% of the world's population living in the developing world rely on medicinal plants as a primary source of healthcare [1]. Rural communities in developing countries mainly depend on medicinal plants due to lack of modern health facilities, cultural priorities, beliefs, cost of modern drugs, and effectiveness of medicinal plants against certain diseases that cannot be cured by modern drugs [2–5]. In developed nations, during the COVID-19 pandemic, the demand for medicinal plants has increased. In a study conducted in several countries, increased consumption of ginger, garlic, onion, turmeric, and lemon as “immune boosters” during the pandemic was reported [6, 7].

Ethiopia is comprised of various climatic zones and consists of 6,000 species of vascular plants of which 10% are reported as medicinal plants (floras). Several studies have been conducted in Ethiopia to document medicinal plants and associated knowledge [8–12], where many of such studies were conducted in Oromia and South Nation Nationalities People Regions [13–15]. In addition, the studies reported that the knowledge on medicinal plants of the country is getting lost due to various reasons. The indigenous knowledge on medicinal plants transfers from generation to generation orally. In this regard, basic information on the use of plants and parts used, methods of drug preparation, and others may be lost in the knowledge transfer system [4]. The expansion of modern education, agricultural expansion, urbanization, overexploitation, and firewood collection were also reported as the main threats to medicinal plants in Ethiopia [16–18].

Basona Werana is one of the districts in the Amhara Region of Ethiopia located in the eastern parts of the Ethiopian Highlands in the Semien Shewa Zone, Amhara Region. All most all inhabitants practiced Ethiopian Orthodox Christianity. The largest ethnic group reported in Basona Werana was the Amhara, and Amharic was spoken as a first language. The top human diseases that are common in Basona Werana District are dyspepsia, typhus fever, pneumonia, diarrhoea, typhoid fever, Helminthiases, tonsillitis, urinary tract infection, and arthritis, while the top livestock diseases in the study area are diarrhoea, sudden disease, stomachache, leech, wound, cough, and rabies. Although there are various health posts in the district, the number of patients and the number of health post are not equivalent. Hence, local communities visit local healers or traditional medicine mainly for the treatment of livestock ailments.

One of the areas where ethnobotanical research is lacking is Basona Werana District. Like other communities living in different parts of Ethiopia, local people living in Basona Werana District use many plant species in human and livestock ailment treatment. However, the knowledge may vanish before a proper documentation as it was evidenced by various studies. Therefore, the main objective of this research was to document medicinal plants and associated indigenous knowledge of local people of Basona Werana District. The study also aimed to assess threats to the medicinal plants of the study area. The findings of this study may serve as a stepping stone for further phytochemical and pharmacological studies.

## 2. Methods

### 2.1. Description of the Study Area

**Figure 1:**
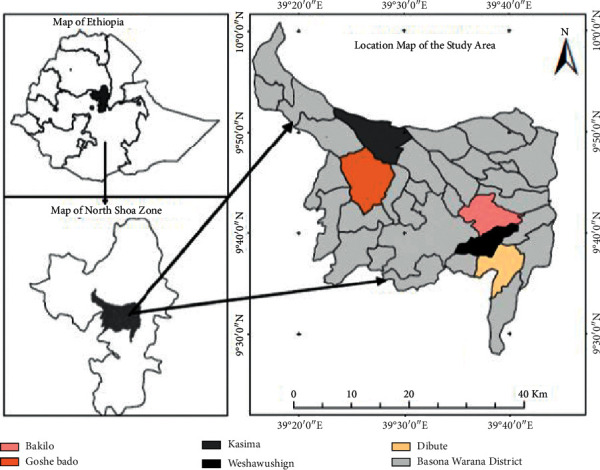
Map of Ethiopia showing Amhara region and the study areas.

Basona Werana is one of the districts in the Amhara Region of Ethiopia located in the eastern edge of the Ethiopian Highlands in the Semien Shewa Zone, Amhara Region (Figure 1). It is 130 km far from the capital city of Ethiopia, Addis Ababa. Basona Werana is bordered on the south by Angolallana Tera, on the southwest by Abichuna Gnea, on the west by Siyadebrina Wayu, on the northwest by Moretna Jiru, on the north by Mojana Wadera, on the northeast by Tarmaber, and on the east by Ankober District.

### 2.2. Climate

**Figure 2:**
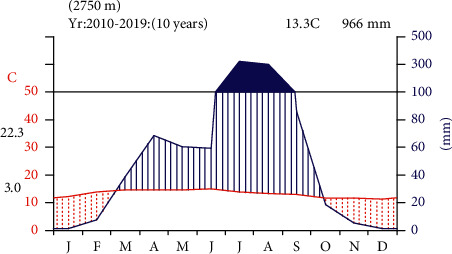
Climadiagram of the study area from 2020 to 2019 at Debre Birhan Station. Data source: National Metrological Service Agency.

In Basona Werana District, the geographical distribution is divided into four agro-climatic zones. These are Dega (50%), Woina Dega (46%), Wurch (2%), and Kola (2%). The annual rainfall of the district is 966 mm, and the mean annual temperature of the district is 13.3 C (Figure 2).

### 2.3. Reconnaissance Survey and Selection of the Study Sites

Basona Werana is a district of Amhara Region of Ethiopia. A reconnaissance survey was conducted from January 30 to February 15, 2020, to select five kebeles based on the availability of traditional medicine history, practitioners, availability of medicinal plants, altitudinal variation, and distribution of climatic conditions between kebeles. Based on this information, five kebeles were selected. These are Weshawushign, Dibute, Bakilo (highland), Goshe Bado (midland), and Kasima (lowland) (Figure 1).

### 2.4. Selection of Informants

A total of 80 informants were selected for ethnobotanical data collection from each study site. Of which, 60 (46 men and 14 women) were nontraditional healers, whereas 20 (16 men and 4 women) were traditional healers. Informants were selected randomly, while traditional healers were selected purposefully and considered as key informants. As pointed out by [19], the selection of key informants is commonly purposive. The age of the informants included in the study ranged from 25 to 70. About 31 informants were aged 56–70, which accounted for 29% followed by 41–55 accounting for 27 (34%), and 27% of informants were aged 25–40. The educational levels of the informants were from illiteracy to college level. Most of the informants were illiterate (53, 66%) followed by elementary school (20, 25%). High school and college-level education accounted for 6% and 3%, respectively.

The selection of key informants was based on the recommendations of knowledgeable elders, religious leaders, kebele administrators, literate people, and personal observations of the researcher from the community group. After that, the key informants were identified, later on, interviewed, and followed for further detail. The selection of key informants was made by asking different questions to traditional herbalists who gave different medicines for human and animal ailments.

### 2.5. Ethnobotanical Data Collection

Ethnobotanical data collection was conducted from February 20 to April 30, 2020, to collect information from the informants. A semi-structured interview was the main data collection tool used during the study. A list of questions based on the objective of the study was prepared in English and translated into Amharic local language of the study area. During the interview, information on vernacular name of the medicinal plants, type of disease treated, parts of the plant used, methods of preparation, mode of administration, conservation practices, and use other than medicinal value was recorded. In addition to semi-structured interviews, data were collected through group discussions and guided field walks with key informants for field observations.

**Figure 3:**
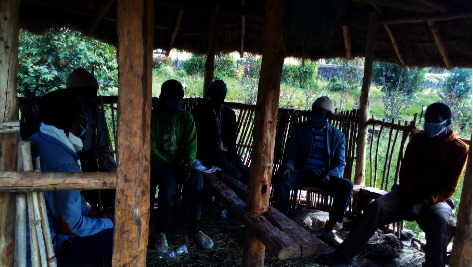
Group discussion with key informants about existing threats to medicinal plants.

The discussion was conducted with 5 to 10 key informants, mainly focusing on threats to medicinal plants, methods of conservation, and how knowledge is maintained and transferred from one generation to another generation (Figure 3).

**Figure 4:**
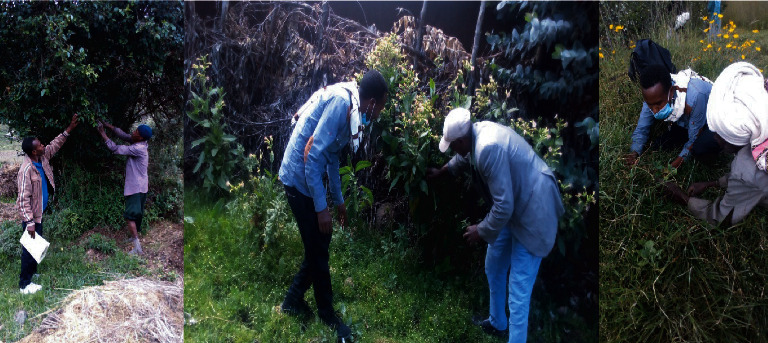
Field observation with the guidance of local people in Basona Werana District.

Field observation with interviews was also conducted in both the wild and the home gardens of the study sites to collect the voucher specimens (Figure 4).

### 2.6. Specimen Collection and Identification

Medicinal plants were collected (from wild and cultivated areas), pressed, and dried for identification. For some species, preliminary identification was conducted in the field. In addition, further identification was done using various volumes of the flora of Ethiopia and Eritrea. After that, the specimens were taken to Madda Walabu University Mini Herbarium.

### 2.7. Ethnobotanical Data Analysis

The collected data about medicinal plants were entered into an Excel spreadsheet 2010 and summarized using descriptive statistical methods such as frequency and percentages. On top of that, informant consensus factor, preference ranking, and direct matrix ranking were used according to [19, 20].

#### 2.7.1. Preference Ranking

Preference ranking was conducted following the recommendation by [19, 20] for the most preferred medicinal plants used to treat stomachache. For this activity, ten informants were selected. Each informant was provided with the mentioned medicinal plants reported to cure the illness with the leaves of the medicinal plants used being paper tagged and then was asked to assign the highest value (6) for the most preferred species against the illness and the lowest value (1) for the least preferred plant. The value of each species was summed up, and the rank for each species was determined based on the total score.

#### 2.7.2. Direct Matrix Ranking

Based on [19, 20], a direct matrix ranking was performed to compare the multipurpose use of a particular species and set this in relation to the extent of its use. To carry out this activity, 10 key informants were selected according to their response during the interview and asked to assign usage values (5 = best, 4 = very good, 3 = good, 2 = less used, 1 = least used, and 0 = not used). Accordingly, each key informant, use values for the multipurpose medicinal plant species, and average value of each use diversity for a species are summed up and ranked.

#### 2.7.3. Informant Consensus Factor (ICF)

The ICF was calculated to assess the reliability of the information and identify the informant's response to the cure of the reported disease category according to the formula [21]. The ICF was calculated as follows: the number of citations used for each disease (nur) minus the number of species used for that disease (nt) is divided by the number of citations used for each disease minus one to obtain the following formula:

(1) $$\begin{array}{c} \text{ICF}=\frac{\text{nur}-\text{nt}}{\text{nur}-1}, \end{array}$$

where nt = number of species used and nur = number of citations used for each ailment.

## 3. Results

### 3.1. Medicinal Plants Collected from the Study Area

A total of 76 medicinal plant species were collected and identified, with 75 genera and 45 families represented. Lamiaceae was the most used plant, with 8 (10.5%) species, followed by Asteraceae with 7 (9%) species and Solanaceae with 6 (7.8%) species.

**Figure 5:**
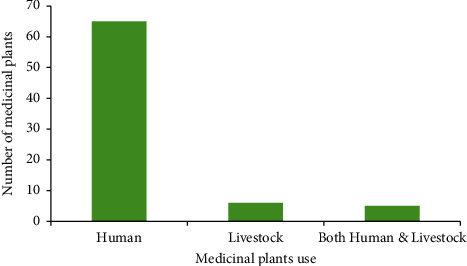
Number of medicinal plants used for human and livestock treatment.

Of the 76 species of medicinal plants collected from the study area, 56 (73.6%) species of them were obtained from the wild, whereas 14 (18.4%) species were from both wild and home garden and only 6 (8%) species were collected from home garden (Table 1). Of 76 medicinal plant species, 65 (85.5%) species were claimed to treat human health problems; 6 (8%) species were claimed to treat livestock ailments; and 5 (6.5%) species were for both human and livestock ailments (Figure 5).

### 3.2. Growth Form and Plant Parts Used to Treat Diseases

**Figure 6:**
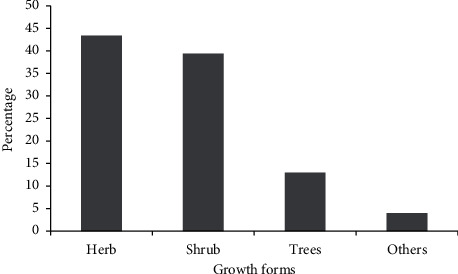
Life forms of medicinal plants collected from Basona Werana District.

Herbs are represented by 33 species (43.4%), shrubs by 30 species (39.4%), and trees by ten species (13.1%), according to the growth form analysis of medicinal plants (Figure 6).

**Figure 7:**
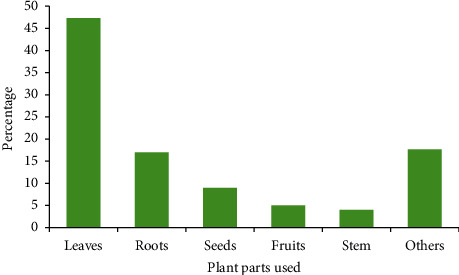
Plant parts used in traditional medicine preparation.

The informants in the study area stated that leaves were the most commonly used plant part for remedy preparation in the study area, accounting for 53 (47.3%) preparations, followed by roots and seeds, which accounted for 19 (16.9%) and 10 (8.9%) preparations, respectively (Figure 7).

### 3.3. Preparation Methods, Condition, and Route of Administration of Traditional Medicine

Crushing was the most common type of traditional medicinal plant preparation, accounting for 38 preparations (33.9%), followed by pounding (18 preparations, 16%) and powdering (11 preparations, 9.8%), respectively. The remaining traditional medicinal preparation methods were organized as others, which accounted for 28% (Table 2).

Local communities in the study area employ medicinal plants in fresh, dried, and fresh or dried forms to make traditional herbal preparations. Fresh forms of medicinal plants were reported to be utilized the most (69.9%), followed by dried forms (17%) and fresh and dried forms (13.1%), respectively.

**Figure 8:**
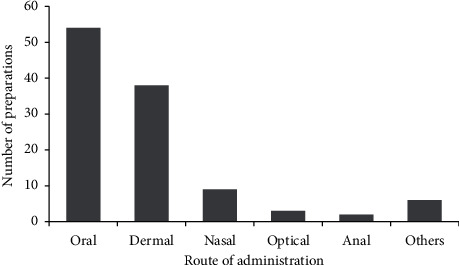
Route of administration of traditional medicine in the study area.

Traditional medicine is usually administered orally in the study area. Oral accounts for 54 (48.1%) of the total, with dermal accounting for 38 (33.9%) and nasal accounting for 9 (8%) of the total (Figure 8).

### 3.4. Dosage and Antidote

Most traditional healers have utilized spoons, coffee cups, and tea glasses in the study area and have inserted their fingers into those tools to guess the necessary dosage. Counting the parts of the plants they used is one of the other approaches. The treatment is usually taken several times until the ailment is cured. Some traditional medicine preparations have been documented to produce diarrhoea and vomiting as side effects. Traditional healers give antidotes for patients to counteract these negative effects. Milk, honey, coffee, and tella (local beer) were given to counteract the overdose effect.

### 3.5. Ranking of Medicinal Plants

Ten traditional healers participated in the preference ranking, and *Cucumis ficifolius* stood first as it is the best preferred medicinal plant used to treat stomachache followed by *Achyranthes aspera* L. and *Cymbopogon citratus* (DC.) Stapf., respectively. *Artemisia abyssinica* Sch. Bip. ex A. Rich. was the least preferred medicinal plant for the treatment of stomachache (Table 3).

### 3.6. Direct Matrix Ranking

Based on the information gathered from the informants, six multipurpose plant species were selected randomly listed as all medicinal plants and the key informants assessed their relative importance used in their localities. With a score of 17 and 11 points, *Croton macrostachyus* Hochst. ex Del. was found to be the most versatile traditional medicinal plant, while *Citrus x limon* (L.) Burm F. was found to be the least multipurpose traditional medicinal plant (Table 4).

### 3.7. Informant Consensus Factor (ICF)

**Figure 9:**
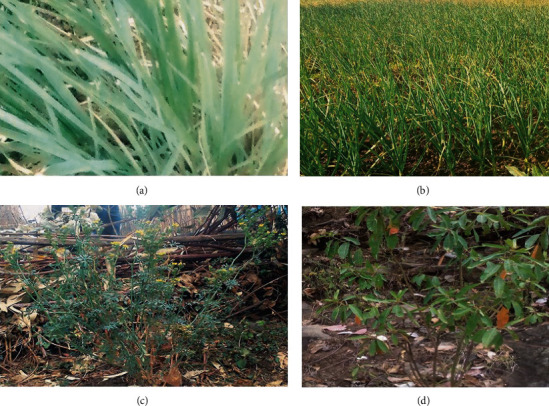
Frequently used medicinal plants for various disease treatments. (a) *C. citratus*. (b) *A. sativum*. (c) *R. chalepensis*. (d) *C. abyssinica*.

In the study area, a total of 54 different types of diseases were found and categorized into eight different groups (Table 5). Dermatological disease (0.78) was the category with the highest ICF value, followed by gastrointestinal tract disease (0.77). In dermatological disease, most plant species (35 plant species) were employed. For instance, *A. sativum*, *R. chalepensis*, *C. abyssinica,* and *C. citratus* were frequently reported for use in respiratory organ-related disease, stomachache, and dermatological diseases (Figure 9).

### 3.8. Threats to Medicinal Plants in the Study Area

According to informants, medicinal plants are primarily threatened by human activity. Agricultural expansion, firewood collection, and charcoal production are the most threatening factors for medicinal plants in the study area (Table 6). Currently, medicinal plants are not easy to harvest and traditional healers have to travel long distances to collect them. *H. abyssinica* (Bruce ex Steud.) J.F. Gmel. and *C. africana* are being harvested for timber production; and *O. europaea* subsp. *cuspidata*, *O. rochetiana, O. lanceolata,* and *C. spinarum* for charcoal and firewood.

### 3.9. Conservation of Medicinal Plants

In the study area, there were plant species that have multiple purpose use. However, local communities of Basona Werana District have no or exert little effort to conserve medicinal plants. Plant species such as *A. sativum, L. sativum, R. chalepensis,* and *Z. officinale* were the frequently grown medicinal plants in home garden.

## 4. Discussion

### 4.1. Diversity of Medicinal Plants

Seventy-six medicinal plants distributed to 75 genera and 45 families were identified and recorded in this study. Compared with the previous studies conducted in Ethiopia, this study reported high and low numbers of medicinal plant species. For instance, [9] collected 266 plant species used by communities of Sheka Zone for the treatment of human and livestock ailments. In a similar study, [16] collected 112 medicinal species, from Yilmana Densa and Quarit districts of Amhara region. The two authors reported a higher number of medicinal plants compared with this study. However, [8, 22, 23] reported 35, 51, and 63 species of medicinal plants, respectively. Vegetation type of the district, number of informants involved in the study, data collection time, and duration and culture could be the reason for the difference in the number of medicinal plants.

This study revealed that the family Lamiaceae has contributed the highest medicinal plant diversity, followed by Asteraceae and Solanaceae. These families are among the top plant families with contributing the largest medicinal plant species as reported from other parts of Ethiopia [8, 9, 18, 24] and elsewhere in the world [25, 26].

Of the 76 species of medicinal plants collected from the study area, the majority of them (56 (73.6%)) were obtained from the wild habitat. This finding is evidenced in other similar studies as medicinal plants are harvested mainly from wild habitats than home gardens [9, 27, 28]. Plant species grown in wild habitat are under pressure from various anthropogenic factors [29].

### 4.2. Growth Forms and Plant Parts Used for the Preparation of Traditional Medicine

Herbaceous species accounted for 33 (43.4%) of the therapeutic plants collected in the Basona Werana District. This may be due to the fact that herbs are more readily available and plentiful in the surrounding areas than shrubs and trees [18]. This result is consistent with the general trend of herbaceous species dominance found in numerous ethnobotanical studies conducted in Ethiopia [4, 8, 16, 18, 30, 31]. In contrast to the present finding, shrubs or trees were the dominant life forms as a contributor of medicinal plants in other findings [11, 17, 28, 32]. The predominance of shrubs or trees over other forms of growth may be due to their annual availability and their relative ability to withstand drought, which may be useful for widespread use [33].

Local people of Basona Werana District used various plant parts for the preparation of traditional medicine. This study indicated that leaves were the most commonly used part of the plant. Similar studies list leaves and roots as the most common parts of plants used to prepare medicines. The studies that reported the leaves as the most utilized plant parts are [5, 8, 12, 30, 32]. The studies that reported the dominance of roots over other plant parts are [24, 28, 34]. Leaves are preferred over other plant parts due to their ease of availability and ease of medication preparation. Furthermore, secondary metabolite storage is beneficial to the medicinal plant's biological characteristics [5]. Because roots and bark take longer to recover than leaves, harvesting them enhances a plant's vulnerability [35].

### 4.3. Preparation Methods, Condition, and Route of Administration of Remedies

Crushing is the most common preparation method in the study area. Crushing as the most common type of preparation is also reported by [8, 18, 28] in different parts of Ethiopia. However, in a similar study on people of Yilmana Densa and Quarit districts, [16] reported that splicing was a dominant method of preparation of remedy. Pounding was the dominant method of traditional medicine preparation by local people of Melokoza District [11]. Most of the remedies from dried parts were prepared by pounding, while the remedies from freshly harvested parts were prepared by crushing [16].

The majority of medicinal plants (69.9%) were found to be used in fresh form in this study. Kassa et al. [9, 18, 36, 37] all reported using freshly collected plant parts for traditional medicine preparation. People's reliance on fresh plant parts is frequently owing to the efficacy of fresh plant species in therapy, as the components are not lost before application, as is the case with dried plant forms [5]. Because local people took limited efforts in conserving dried plant matter, a frequent gathering of fresh plant parts may endanger the plants, especially during dry seasons. The dependency of peoples on fresh plant parts is often due to the effectiveness of fresh plant species in therapy as the ingredients are not lost before practice related to the dried plant forms [5]. Using fresh plant parts can threaten plants by frequent collection, even in the dry season, as locals minimize efforts to store dried plant material for later use [4, 9].

In Basona Werana District, traditional medicine is usually administered orally, which accounts for 48.1% of the total. In similar studies, other researchers reported oral administration as the leading route of application of traditional medicine [8, 17, 30].

### 4.4. Dosage Determination and Antidotes

Local communities use different methods to determine the dose. Spoons, coffee cups, tea glasses, and inserting a finger and counting the parts of the plants are among the approaches used to determine the dosage. This finding is consistent with the report of [9]. A lack of consistency regarding the dosage of medicines to be used was observed among the informants during the interview. It was reported that the lack of precise dosage is one drawback of traditional medicinal plants [38]. Lack of precise dosage will have an impact on the patient as taking overdosage may have many side effects. When such things happen, local healers counteract the side effects by giving milk. A similar finding was reported by [9].

### 4.5. Ranking of Medicinal Plants

The study revealed that all medicinal plants are not equally important for treating ailments. Local communities prefer one over another mainly based on efficacy and availability. In the preference ranking, *C. ficifolius* was the most preferred and ranked medicinal plants used to treat stomachache. In a similar study conducted in Sheka Zone of Ethiopia [9], *C. macrostachyus* was reported to be the most preferred medicinal plant against gastrointestinal problems. Similar finding for Hawassa Zuria showed that the most preferred medicinal plant reported to treat abdominal stomachache was *Eucalyptus globulus* [36]. *A. sativum* and *Z. officinale* were the most preferred medicinal plants for treating common colds in humans [39]. The various findings in different parts of Ethiopia indicate that local communities in their locality have their own preferred medicinal plants against different human and livestock ailments.

The direct matrix ranking indicated that *C. macrostachyus* and *C. africana* ranked first and second in the study area as a multipurpose plant species, respectively. *C. macrostachyus* was reported to be used mainly as medicine, firewood, and fencing. On the other hand, *C. africana* reported mainly for its use in construction and furniture. A various groups of researchers in Ethiopia reported different medicinal plants as a multipurpose plant species. For instance, [36] reported as *Ensete ventricosum* (Welw.) Cheesman was the most preferred medicinal plant used for various purposes by the local people of Hawassa Zuria District. The authors also reported that the plant was used as a type of food, as fodder, in house construction, and for making of a robe. In a similar study, [12] reported that *Warburgia ugandensis* Sprague was used by Guji Oromo of Ethiopia for multiple purposes such as charcoal production, for construction, and furniture. In other studies, conducted in Adwa District of Tigray Region, *O. europaea* subsp*. cuspidata* are regarded as multipurpose plant species mainly used for charcoal production, construction, and as a food [40]. The most commonly used plants are most threatened in the absence of proper conservation, management, and sustainable use measures [9]. Hence, additional conservation measures are urgently needed to prevent the disappearance of these multipurpose plant species [40].

### 4.6. Threat and Consetion of Medicinal Plants

According to the responses from key informants, the main causes of medicinal plant loss in the study area were agriculture expansion, firewood collection, overgrazing, and drought. Other researchers reported a similar finding to this study where agricultural expansion was the most threat to medicinal plants of Damot Woyde District [39], Adwa [40], and Mojana Wodera [41]. The other reported threats to the medicinal plants of Ethiopia were climate change and the spread of invasive species. In Yalo District, the widespread of invasive species such as *Prosopis juliflora* (SW.) DC. is replacing the plants with cultural values and changing vegetation to monotype bushes and forests [42].

The conservation of medicinal plants in the study area was minimal; rather, the utilization of leaves for the preparation of traditional medicine may have some contributions to the conservation of medicinal plants as also reported by another study [4, 36]. In addition, traditional beliefs are also reported to have their own unintentional role in the conservation of medicinal plants [43]. The identification of various threats by current studies and other studies conducted in Ethiopia has shown that different conservation approaches are needed to save medicinal plants from further loss.

## 5. Conclusions

The results of the study revealed that communities of Basona Werana District use a number of medicinal plants for the treatment of human and livestock ailments. A total of 76 medicinal plants were recorded in this study where Lamiaceae was the highest contributor of medicinal plants. Leaves were the predominantly used plant part for the preparation of remedies. If the remedy is overdosed, local communities in the study area use antidotes such as milk and honey. This study also revealed that *C. ficifolius* is the most preferred medicinal plant in the study area used to treat the stomachache. *H. abyssinica*, *C. africana*, *O. europaea* subsp*. cuspidata*, *O. rochetiana, O. lanceolata,* and *C. spinarum* are the most threatened medicinal plants in the area. Thus, we recommend that local communities should apply conservation efforts to protect medicinal plants from further loss. A detailed phytochemical and pharmacological experiment is also recommended on the most preferred medicinal plants for future research in searching of modern drugs.

## Figures and Tables

**Table 1:** List of traditional medicinal plants used to treat human and livestock ailments in Basona Werana District.

Scientific name	Family	Local name (CN)	GH	Habitat	Part used	CD	Health problem treated	Route of application	Used for	Mode of preparation of the remedies
*Achyranthes aspera* L.	Amaranthaceae	Teleji (NT 66)	H	Wild	Root	D	Stomachache	Oral	Human	Root of *A. aspera* with *Rumex nepalensis* Spreng. powdered and mixed with water and then drunk
					Leaf and stem	F	Wound	Dermal	Both	Crushed the leaf and stem and then creamed repeatedly on the affected dermal part
					Leaf	F/D	Skin rash	Dermal	Human	Crushed and tied on the dermal part

*Acmella caulirhiza* Del.	Asteraceae	Yemider berbere (NT 67)	H	Wild	Leaf	F	Toothache	Oral	Human	Shoot apex are taken from *Olinia rochetiana* and *A. caulirhiza* and then pounded and placed on pain tooth

*Acokanthera schimperi* (A.DC.) Schweinf.	Apocynaceae	Merenze (NT 28)	Sh	Wild	Root	D	Rabies	Oral	Livestock	Pounded and mixed in water and then drunk

*Aeonium leucoblepharum* Webb ex A. Rich.	Crassulaceae	Tibitiba (NT 34)	H	Wild	Root	F/D	Rheumatism	Dermal	Human	*A. leucoblepharum* and *R. nepalensis* roots are crushed and applied to the affected part

*Ajuga integrifolia* Buch. Ham.	Lamiaceae	Armagusa (NT 70)	H	Wild	Root	F/D	Herpes	Dermal	Human	Crushed the root and leaf and then heated on fire and placed on the affected body part
					Root	F	Hypertension	Oral	Human	Squeezed or boiled and drunk

*Allium sativum* L.	Amaryllidaceae	Nech shinkurt (NT 13)	H	Home garden	Bulb	D	Influenza virus	Oral	Human	The fruit juice of *Citrus x aurantiifolia* (Christm.) Swingle mixed with the crushed bulb of *A. sativum* and then drunk until recovery
					Bulb		Asthma	Oral and nasal	Human	The bulb with rhizome of *Zingiber officinale* boiled in water, then the filtrate is drunk, and the nasal is fumigated by its vapour

*Aloe debrana* Christian	Asphodelaceae	Eret (NT 41)	Sh	Wild	Leaf	F	Hemorrhoids	Anal	Human	Tied *A. debrana's* jelly on the affected area

*Artemisia abyssinica* Sch. Bip. ex A. Rich.	Asteraceae	Chikugn (NT 73)	H	Wild or home garden	Root	F/D	Diarrhoea	Oral	Livestock	The root and leaves of *A. abyssinica* are crushed and mixed with water and then drenched in goat and sheep
					Root and leaf	F	Diarrhoea	Oral	Both	The fresh root and leaves are crushed and mixed with water and then drunk
					Root	F	Stomachache	Oral	Human	The root of *A. abyssinica, Verbena officinalis, and C. ficifolius* pounded with *A. sativum* bulb and *Lepidium sativum* L. seed and then mixed with water and drunk

*Asparagus africanus* Lam.	Asparagaceae	Yeset-qest (NT 11)	Sh	Wild	Root	F/D	Bleeding after delivery	Fumigation	Human	Mix the roots of *A. africanus, Carissa spinarum, Clerodendrum myricoides* (Hochst.) R. Br. ex Vatke, and *Capparis tomentosa* Lam. and then fumigate the body

*Berberis holstii* Engl.	Berberidaceae	Zenkila (NT 19)	Sh	Wild	Root	D	Eye disease	Optical	Human	The powder of root mixed with butter and applied to the affected eye

*Brassica nigra* (L.) W.D. K. Koch.	Brassicaceae	Sinafch (NT 74)	H	Wild or home garden	Seed	D	Wound	Dermal	Human	Pounded the seed and mixed with Vaseline/honey and then creamed on the affected part

*Buddleja polystachya* Fresen.	Scrophulariaceae	Anfar (NT 31)	Sh	Wild	Leaf and root	F	Hemorrhoids	Dermal	Animal	Roots and leaves of *B. polystachya* crushed with bean gran, *L. sativum* seed, cottonseed, and Aloe sap and then applied to the affected part

*Calotropis procera* (Aiton) Dryand.	Apocynaceae	Kinbo (NT 5)	T	Wild	Root and leaf	F	Hemorrhoids	Anal	Human	Drop its juice on the affected part

*Carissa spinarum* L.	Apocynaceae	Agam (NT 48)	Sh	Wild	Leaf	F	Diarrhoea	Oral	Human	The leaf was powdered and mixed with *Coffea arabica* L. and drunk
					Root	D	Bleeding after delivery	Nasal or Oral	Human	Mix the roots of *C. spinarum, C. myricoides, and C. tomentosa* and then fumigate

*Cicer arietinum* L.	Fabaceae	Shimbra (NT 76)	H	Wild or home garden	Seed	D	Snakebite	Oral	Human	Bake seed powder with *Sesamum angustifolium* (Oliv.) Engl. and eat

*Citrus x limon* (L.) Osbeck	Rutaceae	Lomi (NT 40)	Sh	Home garden	Fruit	F	Skin fungus	Dermal	Human	Creamed and massaged by its juice on the affected skin continuously until recovery

*Clematis simensis* Fresen.	Ranunculaceae	Azo hareg (NT 57)	Cl	Wild	Root and stem	F/D	Swellings	Dermal	Human	Crashed and tied on the affected part
					Leaf	F	Swelling	Dermal	Human	Pound the leaf and tied on wound
					Stem	F	Hemorrhoids	Dermal	Human	Stem of *C. simensis* heated on fire and hold on the affected area

*Clutia abyssinica* Jaub. & Spach	Peraceae	Fyele fej (NT 68)	Sh	Wild	Leaf	F	Dandruff	Dermal	Human	Pounded and squeezed the leaf and then creamed on the affected part continuously until recovery
					Root	F/D	Anthrax	Oral	Human	The roots of *A. aspera*, Tragia cinerea (Pax) M.G.Gilbert & Radcl.-Sm., *Dodonaea viscosa* subsp. *angustifolia* (L.F.) J.G. West, *C. abyssinica, C. spinarum, Searsia retinorrhoea* (Steud. ex Oliv.) Moffett, *C. ficifolius*, and *Thalictrum rhynchocarpum* Quart. Dill. & A. Rich. mixed with “tela” and then drunk

*Coffea arabica* L.	Rubiaceae	Buna (NT 43)	Sh	Home garden	Seed	D	Wound	Dermal	Both	The seed is crushed and the powder is pasted on the wound

*Cordia africana* Lam.	Boraginaceae	Wanza (NT 29)	T	Wild or home garden	Root and seed	F/D	Involuntary urination in bed	Oral	Human	Root and seed will be pounded together, mixed with honey, and swallowed
					Leaf	F	Fire burn	Dermal	Human	Fired the leaf and the ash will be creamed on the burnt part/wound

*Crinum abyssinicum* Hochst. ex A. Rich.	Amaryllidaceae	Yejib-Shinkurt (NT 06)	H	Wild	Root	F	Rheumatism	Dermal	Human	*C. abyssinicum* root mixed with *A. sativum* and hooted by fire and hold on the affected part or the powder of both of the above will be mixed with Vaseline and creamed on the affected area

*Croton macrostachyus* Hochst. ex Del.	Euphorbiaceae	Bisana (NT 36)	T	Wild or home garden	Leaf	F	Chirt	Dermal	Human	Shoot apex mixed with *Aloe trichosantha*'s A. Berger juice and tied on the affected part
					Bark	F	Tapeworm	Oral	Human	Crushed, pounded, mixed with water, and then drunk

*Cucumis ficifolius* A. Rich.	Cucurbitaceae	Yemidirembuay (NT 32)	H	Wild	Stem	F	Wound	Dermal	Human	Fresh *C. ficifolius* stem fired and hold on wound
					Root and leaf	F	Stomachache	Oral	Human	*C. ficifolius* root and leaf are boiled in water, and then the filtrate is drunk
					Root and leaf	F	Herpes	Dermal	Human	Roasted *C. ficifolius* with spider's web and then pounded and mixed with Vaseline or honey and smeared on infected parts
					Root	F/D	Bloody diarrhoea	Oral	Livestock	Crushed and mixed with some water or milk and then drenched to cattle, goat, and sheep

*Cymbopogon citratus* (DC.) Stepf.	Poaceae	Tejesar (NT 22)	H	Wild	Root and leaf	F	Stomachache	Oral	Human	Boiled root and leaf by water and then drunk its filtrate

*Cynoglossum geometricum* Bak. & Wright	Boraginaceae	Chigogot (NT 16)	H	Wild	Leaf	F	Skin rash (Chifie)	Dermal	Human	Leaves from *O. rochetiana, C. geometricum*, Sheep's horn, and *Lagenaria siceraria* (Molina) Standl. seed grind and then massaged by butter

*Cynoglossum coeruleum* Hochst. ex A.DC.	Boraginaceae	Fkrutena (NT 24)	H	Wild	Root and seed	D	Syphilis	Dermal	Human	Pounded the seed and root and mixed with Vaseline and then creamed on the affected part

*Datura stramonium* L.	Solanaceae	Astenager (NT 07)	H	Wild	Leaf	F	Ear parasites	Ear	Human	Leaf of *Nicotiana tabacum* L. squeezed and then added a few droplet of solution into ear

*Discopodium penninervium* Hochst.	Solanaceae	Ameraro (MT 37)	Sh	Wild	Leaf	F	Skin rash	Dermal	Human	Leaves from *A. integrifolia* and *C. macrostachyus* ground and then massaged on the affected part

*Dodonaea viscosa* subsp. *angustifolia* L.f.	Sapindaceae	Kitkita (NT 62)	Sh	Wild	Leaf	F	Skin rash (chifie)	Dermal	Human	Roasted and powdered the leaf and mixed with butter and creamed the affected part
					Root	F	Stomachache	Oral	Livestock	Grind the root and mix in water and then drench for equine

*Dombeya torrida* (J.F. Gmel) Bamps	Malvaceae	Wulkefa (NT 52)	T	Wild	Leaf	F	Fire burn	Dermal	Human	Squeezed and creamed on the affected part

*Dovyalis abyssinica* (A. Rich.) Warb.	Salicaceae	Koshm (NT 64)	Sh	Wild	Root, leaf, and seed	F/D	Bigunj	Dermal	Human	Fresh leaves, root, and seed are ground together and applied on Bigunj

*Echinops kebericho* Mesfin	Asteraceae	Kebercho (NT 55)	Sh	Wild	Root	D	Evil eye	Nasal	Human	Inhale the smoke

*Eucalyptus Globulus* Labill.	Myrtaceae	Nechbahirzaf (NT 14)	T	Wild or home garden	Leaf	F	Influenza	Nasal	Human	Chopped, boiled, and inhaled
							Common cold	Nasal	Human	Boiled and fumigated
							Foot fungi	Dermal	Human	Collected the younger leaves and massaged/placed them underfoot

*Euclea racemosa* L.	Ebenaceae	Dedho (NT 46)	Sh	Wild	Leaf	F	Skin rash (chifie)	Dermal	Human	*O. rochetiana'*s leaves will be ground/pounded and smeared on infected part
							Sudden disease	Oral	Livestock	Mix the powder in water and drink/drench

*Ficus carica* L.	Moraceae	Beles (NT 49)	Sh	Wild	Leaf	F	Ear infection	Ear	Human	Squeezed the leaves by hand and dropped the juice into the ear canal

*Fragaria x ananassa* Duchesne	Rosaceae	Enjorie (NT 04	H	Wild or home garden	Fruit	F	Coughing	Oral	Human	Boiled its fruits with “Suf” and drunk

*Gymnosporia arbutifolia* (Hochst. ex A. Rich.) Loes.	Celastraceae	Atat (NT 39)	Sh	Wild	Root	F/D	Kidney problem	Oral	Human	The root of *C. macrostachyus* crushed and powdered and then mixed with water and drink

*Gymnanthemum amygdalinum* (Del.) Sch. Bip.	Asteraceae	Grawua (NT 60)	T	Wild	Leaf	F	Ascaris	Oral	Human	Pound with *L. ocymifolia* and drink with water
							Morbidity	Dermal	Human	Crushed leaves of *G. amygdalinum* are mixed with water and washed

*Hagenia abyssinica* Bruce ex Steud.) J.F. Gmel.	Rosaceae	Kosso (NT 50)	T	Wild	Fruit	F	Tapeworm	Oral	Human	After crushed and powdered, mixed with milk and boiled and then drunk
							Nightmare/delivery	Oral	Human	Eat with honey or only itself

*Hypericum quartinianum* A. Rich.	Hypericaceae	Ameja (NT 63)	Sh	Wild	Leaf	F	Stomachache for equine	Oral	Livestock	Crushed the leaves and mixed with water and then drunk

*Pentanema confertiflora* (A. Rich.) Mart. Ort.	Asteraceae	Wonagifit (NT 30)	Sh	Wild	Leaf	F	Toothache	Oral	Human	*A. caulirhiza* and *O. rochetiana* leaves are ground and placed on pain tooth
							Anthrax	Oral	Human	Boiled with bark of *Myrica salicifolia* Hochst. ex A. Rich. and leaves of *Rhamnus prinoides* L'Her. and then drunk
							Jaundice	Dermal	Human	Seven twigs from each of *I. confertiflora and R. nervosus* are taken and then ground and tied on the head for three days
							Eye disease	Optical	Livestock	Powdered and mixed with some water and applied to eye

*Jasminum abyssinicum* Hochst. ex DC.	Oleaceae	Tenbelel (NT 51)	Cl	Wild	Leaf	F	Stomachache	Oral	Human	Seven twigs from each *J. abyssinicum* and *Rydingia integrifolia* and root from *C. ficifolius* are taken and pound and then mixed with water and heated by warmed plough and then drunk

*Lens culinaris* Medik.	Fabaceae	Misir (NT 71)	H	Wild or home garden	Seed	D	Skin rash	Dermal	Human	The seed are ground by teeth before eating food and placed on rash

*Leonotis ocymifolia* Burm. F.) Iwarsson	Lamiaceae	Raskimr (NT 58)	Sh	Wild	Leaf	F	Cough and common cold	Nasal	Human	Boiled fresh leaves with water and then fumigated it or
										crushed the leaves and the juice was inserted into the nostrils
					Root	F	Snake bite	Dermal	Human	Crushed and tied on the affected part of the body

*Lepidium sativum* L.	Brassicaceae	Feto (NT 47)	H	Home garden	Seed	D	Diarrhoea that has blood	Oral	Human	Crushed the seed and mixed with milk and then drunk its solution
					Seed	D	Eye disease	Optical	Livestock	Taken shoot apex from *I. confertiflora, A. sativum,* and *M. salicifoli*a bark and leach and then roosted the mixtures pounded and then inserted in the affected eye

*Maesa lanceolata* Forssk.	Primulaceae	Kelewa (NT 08)	Sh	Wild	Seed	F/D	Skin rash with itching	Dermal	Human	Powdered seed will be mixed with oil and creamed on the skin

*Myrtus communis* L.	Myrtaceae	Barsenet (NT 38)	Sh	Wild	Leaf	F	Wound	Dermal	Human	Leaves of *L. sativum* and *R. chalepensis* pounded together and smeared with Vaseline

*Nicandra physalodes* (L.) Gaertn.	Solanaceae	Atefaris (NT 12)	H	Wild	Leaf	F	Dandruff	Dermal	Human	Leaves are crushed and massaged on the affected part. and placed for a long every day until recovery

*Nicotiana tabacum* L.	Solanaceae	Tinbaho (NT 72)	Sh	Wild or home garden	Leaf	F	Cough	Nasal	Livestock	Powdered and smoked
					Leaf and fruit	F	Leech	Oral	Livestock	Ground and mixed with water and then drenched

*Ocimum lamiifolium* Hochst. ex Benth.	Lamiaceae	Damakessi (NT 21)	Sh	Wild or home garden	Leaf	F	Febrile illness	Oral	Human	Crushed leaves of *Laggera crispata* (Vahl) Hepper & J.R.I. Wood and *Salvia nilotica* Juss. ex Jacq. together drunk with coffee or tea
					Leaf	F	Headache	Oral	Human	Boiled the leaf with *S. nilotica*'s root and then drunk

*Olea europaea* subsp. *cuspidata* (Wall. ex G. Don) Cif.	Oleaceae	Weyra (NT 42)	T	Wild	Leaf	F	Swelling pain	Dermal	Human	Pounded the leaves of *O. europaea, Osyris lanceolata* Hochst. & Stedud., and *Myrsine africana* L. and then tied it on the affected body part.
					leaf	F	Tonsillitis	Oral	Human	The leaf is rubbed and the juice is put on cup and drunk

*Olinia rochetiana* A. Juss.	Penaeaceae	Tife (NT 02)	T	Wild	Leaf	F	Hemorrhoids	Dermal	Human	*O. rochetiana* and *C. simensis* leaves will be powdered and applied to the affected part
					Leaf	F	Swelling	Oral	Human	Shoot apex are taken from *O. rochetiana, O. lanceolata*, *M. africana,* and *O. europaea* subsp. *cuspidata* and pound together and then mixed with water and drunk

*Osyris lanceolata* Hochst. & Stedud.	Santalaceae	Keret (NT 45)	Sh	Wild	Leaf	F	Wound	Dermal	Human	The leaf is crushed and then placed and tied on wound

*Rydingia integrifolia* (Benth.) Scheen & V.A. Albert	Lamiaceae	Tinzut (NT 27)	Sh	Wild or home garden	Leaf	F	Breast pain/cancer	Dermal	Human	Ground with leaf of *C. abyssinica and* then tied on the affected breast part

*Phytolacca dodecandra* L'Her.	Phytolaccaceae	Endod (NT 61)	Sh	Wild	Leaf	F	Rabies	Oral	Human	Pounded the leaf and mixed with *C. arabica and* then drink with a teacup every morning until recovery
					Leaf	F	Anthrax	Oral	Human	Shoot is crushed and mixed with water and then drunk

*Plectranthus lactiflorus* (Vatke) Agnew	Lamiaceae	Dibrk (NT 53)	H	Wild	Leaf	F	Diarrhoea	Oral	Human	Roots and leaves of *P. lactiflorus* are crushed; mixed with water and the filtrate is drunk

*Rhamnus prinoides* L'Her.	Rhamnaceae	Gesho (NT 33)	Sh	Wild	Leaf	F	Toothache	Oral	Human	Hold the leaf by the infected teeth during the feeling of ache

*Rosa abyssinica* R.Br.	Rosaceae	Kega (NT 35)	Sh	Wild	Fruit	F	Hypertension	Oral	Human	Powdered, mixed with water, and drunk

*Rumex abyssinicus* Jacq.	Polygonaceae	Mekmeko (NT 44)	H	Wild	Root	F/D	Hypertension	Oral	Human	Mix its powder with milk and then drink

*Ruta chalepensis* L.	Rutaceae	Tene adam (NT 59)	H	Home garden	Root, leaf, and stem	F	Stomachache and common cold	Oral	Human	The fruit, stem, and leaves are boiled together in water then drunk and fumigate

*Salvia nilotica* Juss. Ex Jacq.	Lamiaceae	Hulegeb (NT 54)	H	Wild	Leaf	F	Nose bleeding	Nasal	Human	Massage/grind the leaf and insert in nose
					Leaf	F	Headache	Oral	Human	Crushed the leaves of *S. nilotica* and *O. lamiifolium* together and added them into coffee and drunk
					Leaf	F	Febrile illness	Ear	Human	Leaves of *S. nilotica, C. coeruleum*, and *O. lamiifolium* are mixed together and inserted their juice through ear

*Satureja punctata* (Benth.) Briq.	Lamiaceae	Lomishet (NT 03)	H	Wild	Root	F	Hypertension	Oral	Human	The roots of *S. punctata* and *J. abyssinicum* are pounded and mixed with fresh cow milk and drunk for seven days

*Schinus molle* L.	Anacardaceae	Kundo Berbere (NT 75)	T	Wild	Seed	D	Abdominal pain	Oral	Human	The seed is mixed with *A. sativum* and pounded and drunk

*Sida schimperiana* Hochst. ex A. Rich.	Malvaceae	Chifreg (NT 15)	H	Wild	Root and leaf	F	Wound	Dermal	Human	The leaf and root of *S. schimperiana* are pounded, powdered, and then applied to the affected part

*Solanecio gigas (*Vatke) C. Jeffrey	Asteraceae	Shikoko gomen (NT 69)	Sh	Wild	Leaf	F	Liver disease	Oral	Human	Seven shoot apexes are taken and squeezed into milk and sunflower, juiced, and then drunk

*Solanum americanum* Mill.	Solanaceae	Yayt awut/Etse Eyesus	H	Wild	Leaf	F	Snake bite	Oral	Human	The shoot tips are chewed with leaves of *O. lamiifolium*

*Taraxacum officinale* (L.) Weber ex F.H. Wigg.	Asteraceae	Nechilo (NT 20)	H	Wild	Leaf	F/D	Headache	Nasal	Human	The leaf of *T. officinale* is crushed and sniffed at the sickness time

*Thymus schimperi* Ronniger	Lamiaceae	Tosign (NT 23)	H	Wild	Leaf and seed	F/D	Hypertension	Oral	Human	Leaves and seeds powdered and drunk with tea

*Trigonella foenum-graecum* L.	Fabaceae	Abshe (NT 26)	H	Wild or home garden	Seed	D	Swelling	Oral	Human	The seed is crushed, powdered, mixed with honey and little water, and then boiled like porridge and eaten

*Urtica simensis* Hochst. ex A. Rich.	Urticaceae	Sama (NT 10)	Sh	Wild	Leaf	F	Stomachache	Oral	Human	Boiled in water and drunk

*Verbena officinalis* L.	Verbenaceae	Atuch (NT 09)	H	wild	Root and Stem	F	Diarrhoea and vomiting	Oral	Human	Pounded the leaf, stem, and root and mixed with water then drunk

*Verbascum sinaiticum* Benth.	Scrophulariaceae	Yeahya joro (25)	H	Wild	Leaf	F	Nose bleeding	Nasal	Livestock	Squeezing the leaf and inserting in nose

*Withania somnifera* (L.) Dun.	Solanaceae	Gizawa (NT 56)	H	Wild	Leaf	F	Demon/evil spirit	Oral	Human	Seven twigs/shoot apex are taken from *W. somnifera, L. ocymifolia, and I. confertiflora* and pounded together and mixed with night water or sweet honey and then drunk

*Zehneria scabra* (L.f.) Sond.	Cucurbitaceae	Ybuhie hareg (65)	Cl	Wild	Leaf	F	Amoeba	Oral	Human	Collect leaves and squeeze it and then drink

*Zingiber officinale* Roscoe	Zingiberaceae	Zingble (NT 57)	H	Home garden	Rhizome	F	Common cold	Oral	Human	Boiled *Z. officinale* and drank the filtrate

Key: condition (CD): collection number (CN); growth habit (GH), herb (H), shrub (Sh), tree (T), climber (Cl), fresh (F), dried (D).

**Table 2:** Method of traditional medicinal preparations in Basona Werana District.

No.	Ways of remedy preparation	Number of preparation	%
1	Crushing	38	33.9
2	Pounding	18	16
3	Powdering	11	9.8
4	Boiling	8	7.1
5	Grinding	6	5.3
6	Others	31	28

**Table 3:** Preference ranking of six selected medicinal plants used for treating stomachache.

Ac	Informant (R1—R10)										Total	Rank
	R1	R2	R3	R4	R5	R6	R7	R8	R9	R10		
*Cucumis ficifolius*	5	6	5	6	6	6	6	3	5	6	54	1st
*Achyranthes aspera*	4	5	4	3	5	5	4	4	6	5	45	2nd
*Cymbopogon citratus*	3	4	6	4	4	4	5	5	2	4	41	3rd
*Ruta chalepensis*	6	3	2	5	3	3	3	6	4	2	37	4th
*Jasminum abyssinicum*	2	2	3	1	1	2	2	1	3	3	20	5th
*Artemisia abyssinica*	1	1	1	2	2	1	1	2	1	1	13	6th

**Table 4:** Average score for direct matrix ranking of six medicinal plant species.

Plant species	Use categories								
	Medicinal	Food	Fodder	Furniture and construction	Firewood	Charcoal	Fencing	Total	Rank
*Citrus x limon*	4	2	0	0	3	0	2	11	6th
*Cordia africana*	2	0	1	5	3	3	1	15	2nd
*Croton macrostachyus*	4	0	1	2	5	2	3	17	1st
*Dovyalis abyssinica*	2	1	3	0	4	1	3	14	3rd
*Ocimum lamiifolium*	5	1	4	0	2	0	1	13	4th
*Olinia rochetiana*	5	1	3	0	1	0	2	12	5th

**Table 5:** Informant consensus factor by category of diseases.

No.	Disease category	No. of plant species	%	No. of use citation	%	ICF
1	Dermatological disease	35	31.8	162	37.5	0.78
2	Gastrointestinal tract	24	21.8	105	24.3	0.77
3	Delicate organ-related diseases	18	16.3	73	16.8	0.76
4	Respiratory organ-related disease	14	12.7	46	10.6	0.71
5	Eye, teeth, and ear infections	9	8.1	22	5	0.61
6	Snake bite and febrile illness	5	4.5	15	3.4	0.42
7	Sexual transmitted diseases and kidney	3	2.7	5	1.1	0.5
8	Evil eye	2	1.8	4	0.9	0.6

**Table 6:** Threats to medicinal plants in Basona Werana District.

Threats	R1	R2	R3	R4	R5	R6	R7	R8	R9	R10	Total	Rank
Agricultural expansion	4	2	3	4	3	4	4	3	4	3	34	1st
Firewood and charcoal	3	3	4	3	4	3	2	4	3	3	32	2nd
Overgrazing	4	3	4	3	2	2	3	2	2	4	31	3rd
Draught	2	1	3	4	2	4	3	3	2	4	28	4th

## Data Availability

The data used in this study are available from the corresponding author upon request.
